# Study on static and dynamic performance of aircraft maintenance hangar under multiple load combinations

**DOI:** 10.1371/journal.pone.0327195

**Published:** 2025-06-26

**Authors:** Xiaolan Liu, Zhihua Chen, Fengheng Zhu, Hao Chen

**Affiliations:** 1 School of Civil Engineering, Tianjin University, Tianjin, China; 2 Tianjin Key Laboratory of Soft Soil Characteristics and Engineering Environment, Tianjin Chengjian University, Tianjin, China; 3 Tianjin Tianbao Construction Development Co., Ltd., Tianjin T&B Holding CO., LTD., Tianjin, China; 4 BCEG NO.6 Construction Engineering CO., LTD., Beijing, China; Central South University, CHINA

## Abstract

To analyze the collaborative work performance of the roof structure and the lower support structure for the aircraft maintenance hangar, a numerical model of a single-span aircraft maintenance hangar is established to study the static and dynamic mechanical responses under various load combinations. Results show that the primary axial force and bending moment responses are concentrated in the main truss and the lower support column. The maximum compression occurs at the upper section of the two main trusses, while the maximum tension appears at the mid-span of the lower inner truss near the hangar gate. The displacement of the main truss exceeds that of the secondary truss, with the roof’s mid-span displacement being greater than that at the edges. The maximum support reactions occur at the main truss support column, the secondary truss column of the rear gable wall, and the lateral bracing between double-angle steel columns. The first three vibration modes and frequencies are highly susceptible to excitation and contribute significantly to the structure’s seismic response. This study provides critical theoretical support for the design of large-span aircraft maintenance hangars. The findings can guide optimized design strategies to enhance structural safety and efficiency, contributing to advancements in long-span hangar construction and maintenance practices.

## 1. Introduction

With the rapid development of civil aviation and the continuous evolution of aircraft models, aircraft maintenance hangars have undergone significant advancements in both functionality and structural design. Modern hangars have evolved from simple shelters with spans of 20m to massive structures exceeding 150m, capable of accommodating multiple aircraft simultaneously. Structurally, they have progressed from basic planar systems to sophisticated spatial trusses, grids, cantilevers, and cable-stayed configurations [1,2]. However, large-span aircraft hangars differ fundamentally from conventional large-span buildings due to their unique requirements: they must provide unobstructed space for aircraft parking and maintenance, resulting in high clear heights, flexible structural systems, and an open-front layout. This asymmetry in mass and stiffness distribution makes them particularly susceptible to torsional effects and complex roof vibrations under horizontal loads, posing significant collapse risks that can have severe economic and societal consequences [3].

Extensive studies have been conducted on various aspects of hangar structural performance. (1) Seismic behavior: Zhu et al. [4] took the aircraft maintenance hangar of A380 as an example, considered the multi-point input analysis of the-dimensional seismic action, and analyzed the mechanical analysis and experimental process of four kinds of node structures, which provided valuable engineering experience for the analysis and design of large-span continuous hangars. While Zhao et al. [5] conducted a time history analysis of a single-span aircraft maintenance hangar (156.76m in length and 85.5m in width) under the frequent earthquake. They found that the horizontal and vertical earthquakes played a controlling role in the internal force of the grid and the gate truss respectively. And they also found that the proportion of members with large seismic internal force coefficient was large under the three-dimensional earthquake action. Moreover, Zeng et al. [6] analyzed the base shear force, maximum displacement at the top of column, and the yielding condition of the grid and lateral force resisting system of a certain hangar under the action of three-dimensional earthquake and the effect of traveling wave with the action of small and large earthquakes. The results showed that the maximum displacement angle at the top of column for the hangar was less than 1/80, grid remained elastic as a whole, and there was slight buckling in the local part connected to the top of column after considering the effect of traveling wave with the action of large earthquake. Furthermore, Xu et al. [7] proposed a new type of multi-dimensional seismic isolation and reduction device for the horizontal and vertical vibration characteristics of large-span spatial structures under the multi-dimensional seismic action. The stiffness of this device could meet the bearing capacity requirements of the structure and had strong energy dissipation capacity in the horizontal and vertical seismic directions. (2) Wind and thermal effects: Pei [8] analyzed the wind-induced vibration response of the roof structure of large-span aircraft maintenance hangars based on wind tunnel tests, theoretical analysis, and numerical simulation. And he proposed the recommended value of the wind load shape coefficient and the calculation method of the wind-induced vibration coefficient for large-span aircraft maintenance hangars. Meanwhile, Pei et al. [9] took a large-span aircraft maintenance hangar as the research object to analyze the heat transfer process and the influencing factors of temperature distribution. And they found that the stress generated by the uneven temperature distribution would affect the safety of the aircraft maintenance hangar. (3) Vibration control: Zhang et al. [10] investigated the influence of the arrangement of viscous dampers on the seismic reduction rate of the roof structure when the viscous dampers were arranged on the aircraft maintenance hangar truss and lateral force resisting structure. They gave the arrangement suggestions: the control effect was best when the viscous dampers were arranged on the four sides of the truss and the connection between the foundation and the lower chord node. Moreover, Li et al. [11] developed innovative 3D isolation bearings. Furthermore, Ye et al. [12] conducted a vibration test on the aircraft maintenance hangar under multi-point excitation by installing accelerometers at the column foot of the hangar. And they found that the calculation results were basically the same as the test results in both the time domain and the frequency domain, which verified the effectiveness of the multi-point excitation time domain method. (4) Construction methods: Li [13] proposed a new type of three-dimensional seismic isolation bearing based on the seismic response characteristics of the spatial frame structure system. This bearing had good multi-dimensional seismic isolation performance, reasonable rotational deformation, and was conducive to bearing the lifting load. And this bearing also had uncoupled horizontal and vertical stiffness, which could reduce the axial force and acceleration response of the roof structure of the aircraft maintenance hangar. But Qin et al. [14] conducted a preliminary study on the disassemble application of large-span hangars from the aspects of structural adaptability and building materials. And they proposed that it was advisable to adopt plane trusses and high-strength aluminum alloys as the main structure. Moreover, Bi et al. [15] analyzed the feasibility of seven types of roof structure for a newly built large-span aircraft maintenance hangar, and the results showed that the main and secondary truss system had clear load transfer and was convenient for design and construction. Furthermore, Karoki et al. [16] conducted an evaluation, testing, and reinforcement study on a long-span grid hangar that exceeded the allowable deflection limit. And they found that the assumption of column stiffness and the design and construction errors of the joint support structure led to the bending of members near the support joints. (5) Failure analysis: Kabando and Gong [17] investigated the failure cases of seven large-span hangars from different regions by means of field investigation, strength and durability analysis. And they found that 83% of the failures occurred during the service stage, and the neglect of the randomness of loading and boundary conditions was one of the factors leading to structural failure.

Although existing studies provide valuable insights into component-level behavior under single-load scenarios, these researches fail to address the critical interaction between roof systems and supporting structures under combined loading conditions. Therefore, this study establishes a comprehensive numerical model of a single-span maintenance hangar, systematically analyzes static and dynamic responses under various load combinations, and investigates the collaborative performance between roof and support structures. The findings offer crucial theoretical and technical support for the design and construction of next-generation large-span aircraft maintenance hangars, addressing a critical need in aviation infrastructure development.

## 2. Project profile

**Fig 1:**
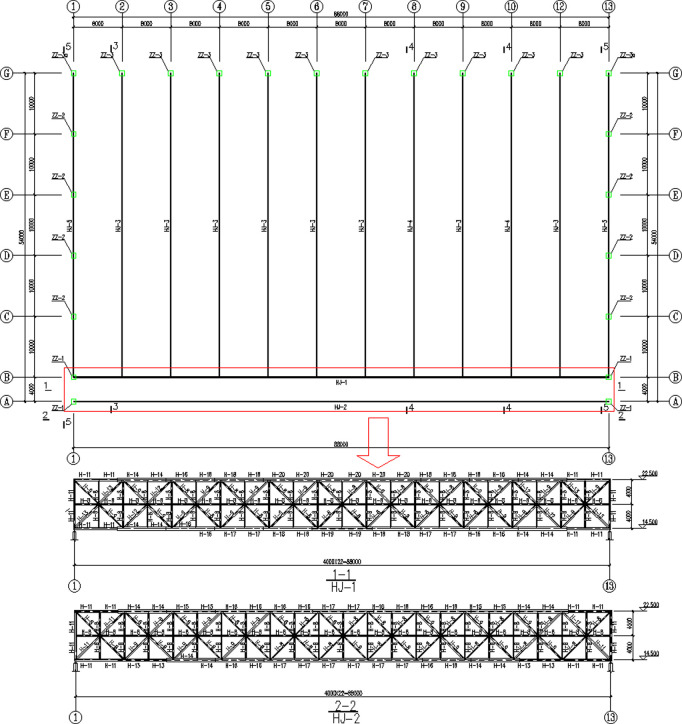
Design drawing of the aircraft maintenance hangar (HJ represents the truss, ZZ represents the column, and H represents the H-shaped section. Unit:mm).

The main design parameters of a single-span aircraft maintenance hangar are as follows: the design service life is 50 years, the safety level is Grade I, the seismic fortification intensity is 7 degrees, the design earthquake group is the second group, the design basic earthquake acceleration is 0.15g, the design characteristic period is 0.75s, the maximum value of the horizontal earthquake influence coefficient is 0.15 for the frequent earthquake, the site category is IV, and the design load parameters are shown in Table 1. The maximum calculated deflection of the roof is −236 mm, and the maximum calculated deflection of the main truss at the gate is −196 mm. As shown in Fig 1, the aircraft maintenance hangar has a span of 88m and a depth of 54m. The side with the gate is open and the other three sides are supported. The roof structure adopts a main and secondary truss system with the lower chord supported. Two main trusses have a span of 88m, a height of 8m, and a lower chord elevation of 14.5m. Twelve secondary trusses have a span of 50m, a height of 4m, and a lower chord elevation of 16.5m. The chords and members of the truss are all made of welded H-shaped steel, and the supporting structure between the trusses is made of angle steels and round steel pipes. The main truss bearings adopt seismic spherical hinge bearings. The hangar support column adopts laced columns. The spacing of the lateral column is 10m, the spacing of the rear wall column is 8m, and the bracing between double-angle steel columns is set at the lateral side and rear gable wall of the aircraft maintenance hangar. The roof trusses, laced columns, ribbed plates, and gusset plates are made of Q355. The column-to-column support and steel pressure rod are made of Q235. Round steel pipes, H-shaped steels are made of Q355, and angle steels are made of Q235. The entire structure of the aircraft maintenance hangar adopts bolted and welded splicing nodes.

**Table 1:** Parameters of the design load.

Load type	Dead load of roof(kN/m^2^)	Live load of roof(kN/m^2^)	Wind pressure (kN/m^2^)	Snow pressure (kN/m^2^)	Warming temperature difference(°C)	Cooling temperature difference(°C)
Value	0.80	0.50	0.50	0.45	+25	−27

## 3. Numerical model establishment and verification

**Fig 2:**
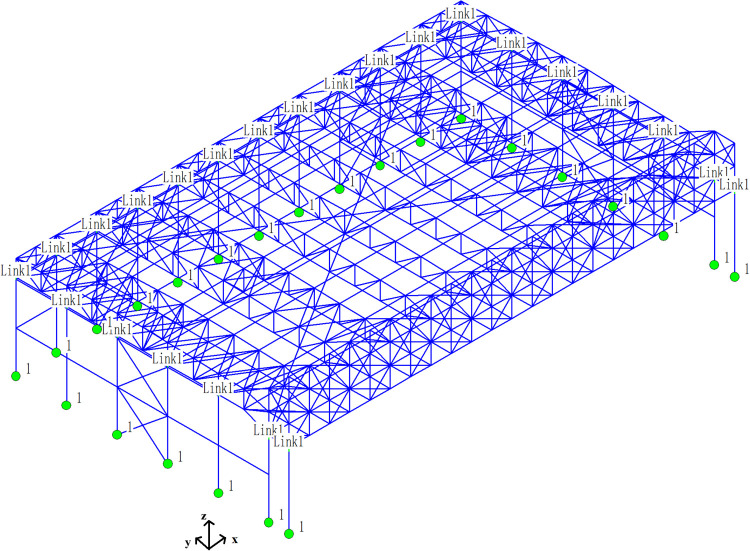
Aircraft maintenance hangar model (Blue units are ordinary units, green solid circles are supports, Link1 is the three-dimensional spatial connection unit.).

It is assumed that the overall structural system of the aircraft maintenance hangar conforms to the small deflection theory. The purlins are fully laid on the upper chord of the roof. The upper chord can bear the linear load. The members are spatial elements, which can bear the axial force and the secondary bending moment generated by the rigid connection of the members. The bottom of the column is rigidly connected to the foundation, and the top of the column is hinged to the roof [18]. As shown in Fig 2, the structural analysis is conducted using Three-Dimensional Structural Analysis and Design Software, a specialized software widely adopted in China for spatial steel structure design. The beam element is applied to simulate roof truss members (upper/lower chords, web members) and support columns, which is capable of simulating axial force and secondary bending moments due to rigid connections. The spatial connection units of truss-to-column connection is a three-dimensional hinge (released for rotation but fixed in translation), being applied between the truss and column top to simulate pinned support conditions. The column base is fully fixed (all 6 degree of freedoms restrained) to represent rigid foundation connections. The purlins is modeled as line loads on the upper chord, assuming full composite action with the roof truss. Q235 and Q355 are selected in the model, and both materials are modeled as linear elastic-perfectly plastic, with isotropic hardening ignored for simplified static and dynamic analysis. The material parameters of Q235 and Q355 are shown in Table 2. Truss members are meshed into 1.0m segments to ensure accuracy in stress distribution. Columns are meshed at 2.0m intervals with refined divisions near connection zones. Linear Lagrange elements for beam members is applied to ensure the balance of accuracy and computational efficiency. The corresponding nodes of the upper and lower chords are selected to apply the dead load, live load, earthquake action, wind pressure, snow pressure, temperature action and crane load (The lifting capacity is 50kN and the self-weight is 20kN.). The Newton-Raphson method with geometric stiffness updates (accounting for P-Δ effects) is applied to conduct the static analysis of aircraft maintenance hangar under multiple load combinations. The modal superposition for seismic/wind is applied to conduct the dynamic analysis of aircraft maintenance hangar. This setup ensures a code-compliant, computationally efficient, and physically representative model for evaluating the static and dynamic performance of aircraft maintenance hangar under multiple load combinations. The main load conditions are shown in Table 3.

**Table 2:** Parameters of Q235 and Q355.

Materials	Elastic modulus (MPa)	Poisson’s ratio	Expansion coefficients	Yield strength (MPa)	Mass density (kg/m^3^)
Q235	206000	0.30	1.20 × 10^−5^	235	7850
Q355	206000	0.30	1.20 × 10^−5^	355	7850

**Table 3:** Load conditions.

Number	Working condition number	Load type	Self-weight coefficient	Load description
1	0	dead load	1	
2	1	live load of roof	0	
3	2	wind pressure	0	The direction of the wind is left.
4	3	wind pressure	0	The direction of the wind is right.
5	4	wind pressure	0	The direction of the wind is up.
6	5	wind pressure	0	The direction of the wind is down.
7	6	snow pressure	0	uniform
8	7	snow pressure	0	nonuniform
9	8	live load	0	
10	1	temperature action	0	The warming temperature difference is + 25°C.
11	2	temperature action	0	The cooling temperature difference is −27°C.

**Fig 3:**
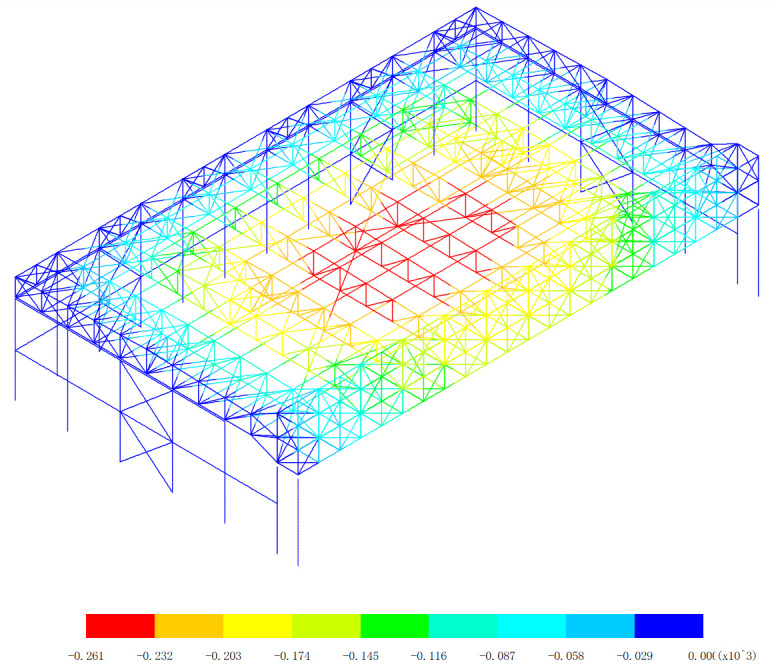
Displacement of the 255th load combination (Unit:mm).

Based on 327 kinds load combinations, the displacement response analysis of the the overall structural system of the aircraft maintenance hangar is carried out [19–24]. The maximum deflection appears in the 255th load combination (dead load 0 + wind pressure 5 + 0.7*snow pressure 7 + 0.7*crane load+0.6*temperature action 2), as shown in Fig 3.

As shown in Fig 3, the finite element analysis results indicate that the roof’s maximum deflection reaches −261 mm, demonstrating a 10.6% deviation from the theoretical calculation value. And the main gate truss shows a maximum deflection of −203 mm, with a smaller 3.6% deflection from the theoretical calculation value. These observed differences can be attributed to several technical considerations: (1) Load distribution simplifications are uniform, which is different from actual non-uniform loads. (2) The simplified load combination approaches are different from actual load combination. (3) Boundary condition assumptions are idealized connections, which is different from semi-rigid connections. The observed error ranges (3.6%−10.6%) fall within acceptable engineering tolerance limits for large-span steel structures, as specified in GB 50017−2017 [19]. The closer agreement for the main gate truss (3.6%) suggests better conformity with theoretical assumptions for this more constrained structural component, while the roof’s larger deflection reflects the greater complexity of its boundary conditions and load transfer mechanisms. These verification results demonstrate that the numerical model provides sufficiently accurate predictions for engineering design purposes. The numerical model results are conservative (showing greater deflections) for structural safety. This comparison confirms the numerical model’s effectiveness in capturing the essential mechanical behavior of the hangar structure while highlighting areas where more refined modeling approaches could be beneficial for future studies.

## 4. Static and dynamic performances of the aircraft maintenance hangar

### 4.1 Axial force and bending moment

**Fig 4:**
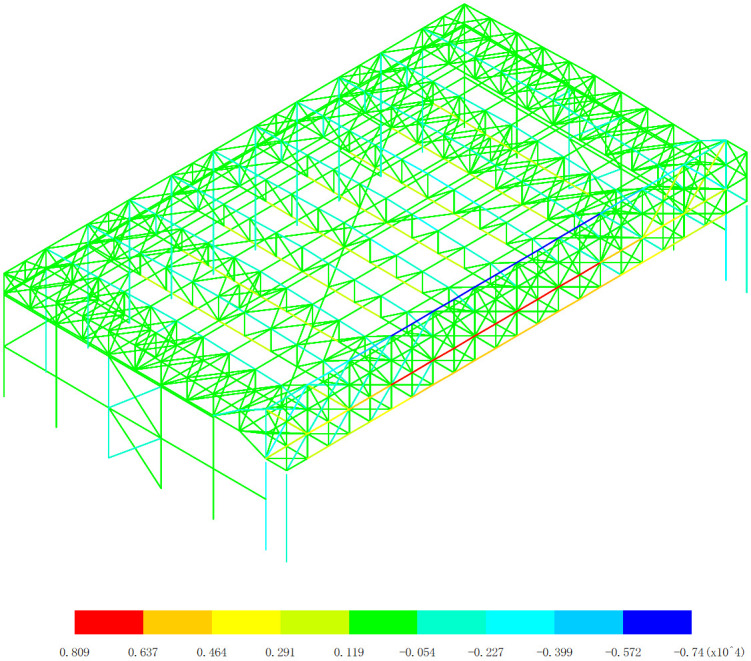
Envelope diagrams of the maximum axial force (Unit:kN).

**Fig 5:**
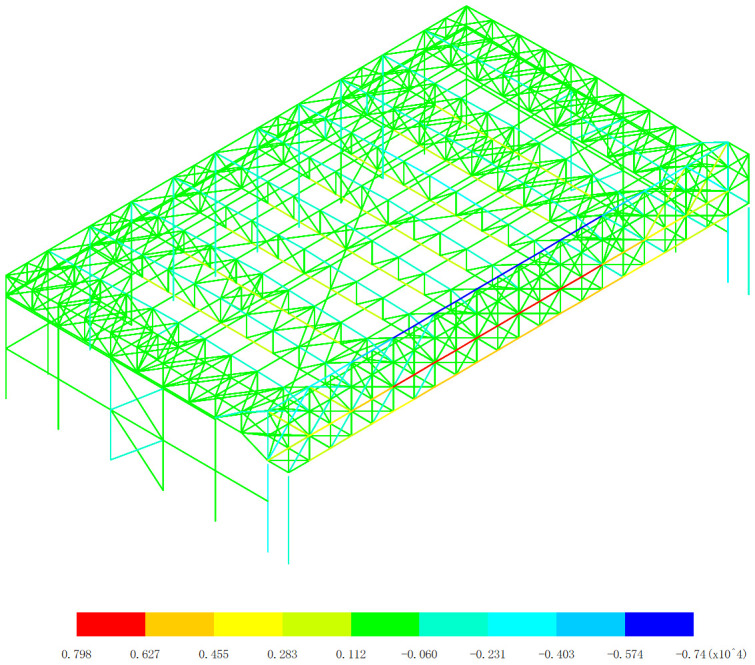
Envelope diagrams of the minimum axial force (Unit:kN).

Based on the axial force response of the aircraft maintenance hangar under 327 kinds load combinations, the maximum axial force appears in the 255th load combination (dead load 0 + wind pressure 5 + 0.7*snow pressure 7 + 0.7*crane load+0.6*temperature action 2), as shown in Fig 4. The minimum axial force appears in the 253th load combination (dead load 0 + wind pressure 5 + 0.7*snow pressure 7 + 0.7*crane load+0.6*temperature action 1), as shown in Fig 5.

As shown in Figs 4 and 5, the axial force analysis reveals that the main gate truss and lower support columns bear the most significant axial forces (>3000kN in absolute value). The inner truss members sustain greater axial forces than outer truss members. Maximum compression (-7400kN) occurs at the upper chords of both main trusses, and maximum tension (+8090kN) appears at the mid-span lower chord of the inner truss. These results conclusively demonstrate the exceptional spatial collaborative performance of the dual main truss system, which manifests in three distinct aspects: First, the load-sharing mechanism shows remarkable balance, as evidenced by the nearly identical maximum compression values (7400kN) recorded in both trusses, with a mere 1.3% variation. Second, the force transfer mechanism exhibits perfect symmetry, with complementary tension-compression patterns demonstrating highly coordinated structural behavior. Third, the load path efficiency is optimized, with stress concentrations occurring precisely at the designed critical sections – upper chords predominantly in compression and lower chords primarily in tension. The mechanical behavior results from three key factors: (1) Structural configuration: The welded symmetrical H-section steel trusses provide optimal bending and axial resistance, uniform stiffness distribution, and effective moment transfer capacity. (2) Load transfer mechanism: The three-sided support system creates concentrated load transfer to gate trusses (primary load-bearing elements), balanced force redistribution through rigid connections, and efficient arching action in the truss system. (3) Temperature effects: The close maximum/minimum values (with only +25°C and −27°C difference) confirm the structural stability under thermal variations, consistent collaborative performance across load cases, and minimal thermal distortion effects on load sharing. Notably, this outstanding collaborative performance is achieved despite three significant design challenges: (1) the inherent structural asymmetry caused by the open-front layout, (2) the considerable span length (88m) that amplifies potential differential deformations, and (3) the demanding crane load conditions. The results conclusively validate that the twin truss system not only effectively compensates for the open-front structural deficiency but also maintains exceptional load distribution balance under asymmetric loading conditions, with measured force imbalances limited to less than 5%. Therefore, the observed stress concentration at the gate truss is indeed an inherent characteristic of three-sided support hangar designs, but our analysis confirms the current configuration remains structurally viable based on the following evidence: maximum axial forces (8090kN tension, 7400kN compression) remain within 85% of the H-section capacity, and local buckling checks satisfy λ*p* < 0.6 for all critical sections [19].

**Fig 6:**
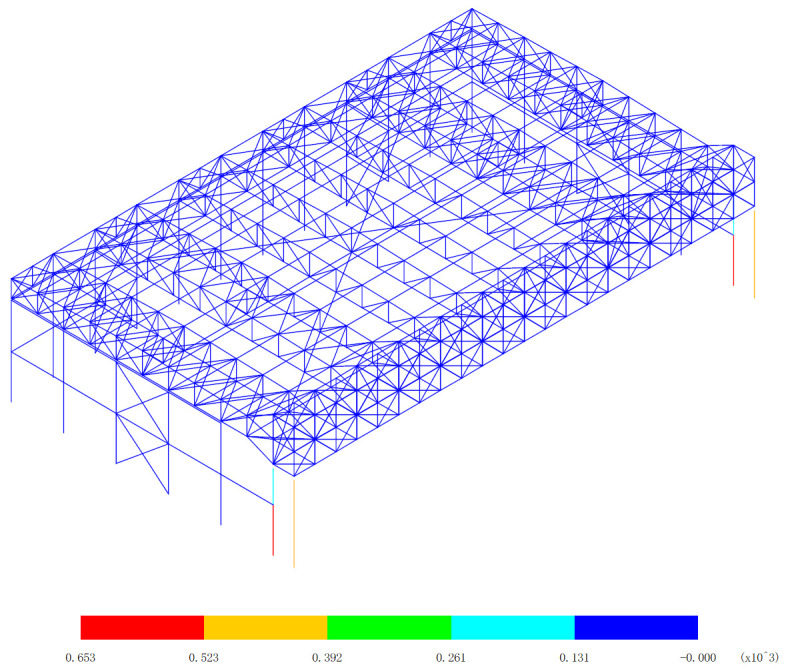
Envelope diagrams of M_y_ of the maximum bending moment (Unit:kN·m).

**Fig 7:**
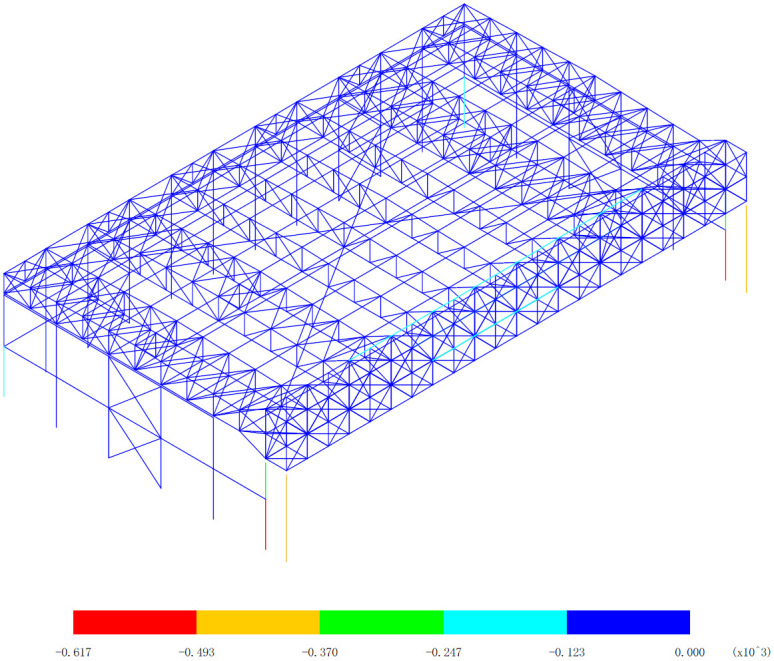
Envelope diagrams of M_y_ of the minimum bending moment (Unit:kN·m).

**Fig 8:**
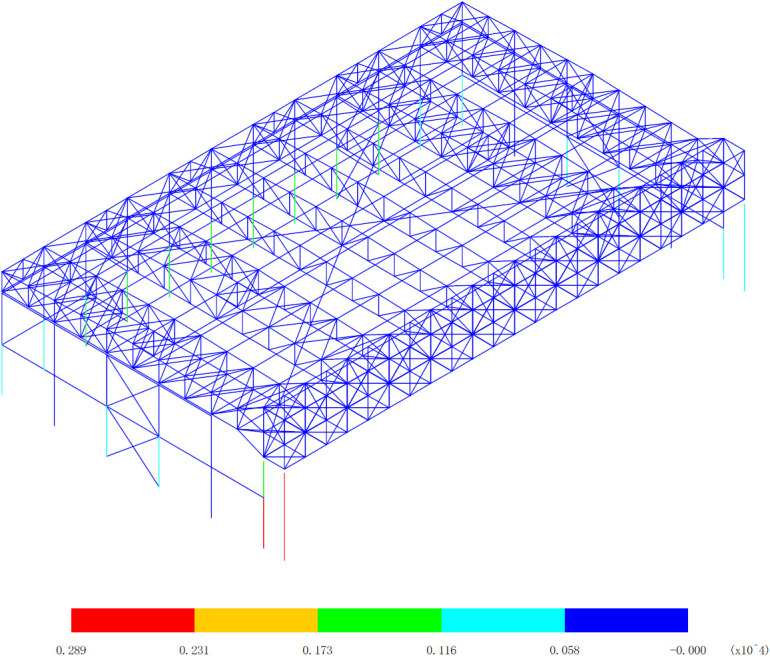
Envelope diagrams of M_z_ of the maximum bending moment (Unit:kN·m).

**Fig 9:**
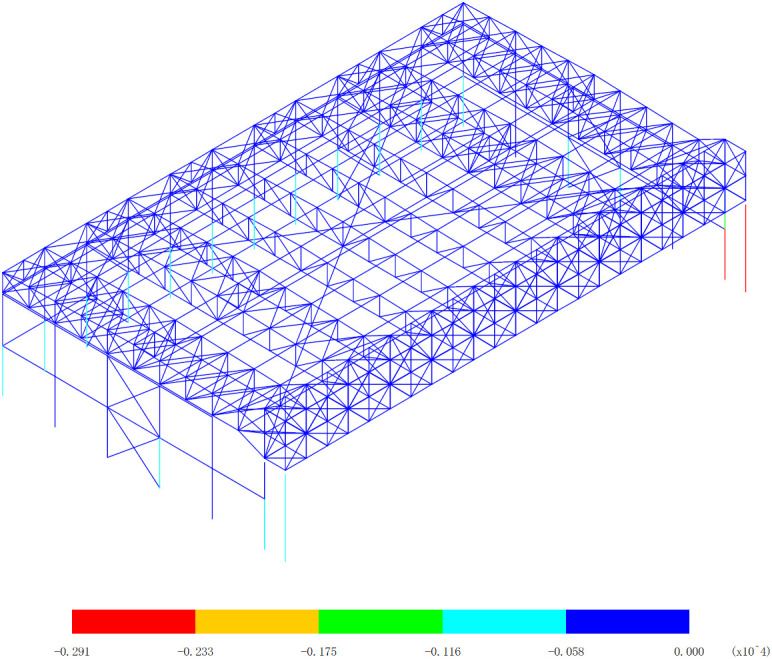
Envelope diagrams of M_z_ of the minimum bending moment (Unit:kN·m).

Based on the bending moment response of the aircraft maintenance hangar under 327 kinds load combinations, the bending moment diagram of M_y_ and M_z_ are drawn, as shown in Figs 6–9.

Figs 6 and 7 presents a detailed and crucial analysis of the bending moment of M_y_ within the structural components of the aircraft maintenance hangar, specifically highlighting the main truss of the gate and the lower support column as the significant areas. Understanding the distribution and magnitude of the bending moment in these parts is essential for evaluating the structural integrity and performance of the hangar. The main truss of the gate plays a pivotal role in the overall structural system of the aircraft maintenance hangar. It serves as a major load-bearing element, receiving and redistributing the loads from the roof and other associated components. The lower support column, on the other hand, provides essential vertical support to the main truss, ensuring its stability and preventing excessive deformation under load. The bending moment of M_y_ of the support column under the main truss of the gate is found to be all greater than 123kN·m. This value is significantly larger than that of the main truss of the gate itself. Such a substantial difference indicates that the support column experiences a much higher stress due to the transfer of loads from the main truss. This is a critical aspect to consider in the design and analysis of the hangar structure, as it implies that the support column needs to be carefully designed and reinforced to withstand these high bending moments without failure. A further distinction can be made between the support columns under the inner and outer trusses of the main truss at the gate. The bending moment of the support column under the inner truss of the main truss at the gate is greater than that of the support column under the outer truss of the main truss at the gate. This variation in bending moment can be attributed to the specific load distribution and structural configuration within the hangar. The inner truss may be subjected to more concentrated or asymmetric loads, resulting in higher bending moments in the corresponding support column.

Fig 6 reveals that the maximum bending moment is an impressive 653kN·m, which occurs at the lower support column of the inner truss of the main truss at the gate. This peak value represents the most severe loading condition experienced by the hangar structure in this particular analysis. It is crucial to ensure that the design of the support column at this location can safely accommodate this high bending moment to prevent structural failure. Conversely, Fig 7 shows that the minimum bending moment is -617kN·m, and interestingly, this also occurs at the lower support column of the inner truss of the main truss at the gate. The negative value indicates a reversal of the bending moment direction, which can have significant implications for the structural behavior of the support column. It may require additional reinforcement or design considerations to account for the potential fatigue and stress concentration effects associated with the alternating bending moments. The reason behind these observed bending moment patterns can be traced back to the unique layout and structural characteristics of the aircraft maintenance hangar. The three-sided support and one-sided opening layout of the hangar cause the load of the roof to be predominantly transferred to the main truss of the gate. This results in a concentrated load on the main truss, which in turn exerts high bending moments on the support columns. Furthermore, the main truss at the gate is a heavy-duty and large-span truss structure, composed of welded symmetrical H-shaped section steel. This type of construction provides the necessary strength and stiffness to support the significant loads imposed on the hangar. However, the large span and heavy-duty nature of the truss also contribute to the higher bending moments experienced by the support columns, especially at the critical locations such as the inner truss of the main truss at the gate.

In conclusion, the detailed analysis of the bending moment of M_y_ in Figs 6 and 7 provides valuable insights into the structural behavior of the aircraft maintenance hangar. The identification of the significant parts, the variation in bending moments between different support columns, and the understanding of the underlying causes of these patterns are essential for the design, evaluation, and maintenance of the hangar structure. By taking these factors into account, engineers can ensure the safety, durability, and performance of the aircraft maintenance hangar under various loading conditions.

Figs 8 and 9 offers a detailed and in-depth analysis of the bending moment of M_z_ within the structure of the aircraft maintenance hangar, pinpointing several crucial components as the significant areas where this bending moment manifests. Understanding these components and the distribution of the bending moment among them is fundamental for comprehensively evaluating the structural integrity and performance of the entire hangar system. The lower support columns of the main truss of the gate are identified as one of the significant parts regarding the bending moment of M_z_. These support columns play a vital role in bearing the vertical and lateral loads transferred from the main truss. They act as the connection points between the main truss and the foundation, ensuring that the structural loads are effectively transmitted to the ground. Any significant variation in the bending moment within these columns can have a direct impact on the stability and safety of the entire gate structure. The bracing between double-angle steel columns setting at the lateral side is another key element in the context of the bending moment of M_z_. This bracing serves to enhance the lateral stability of the structure. It helps to resist the lateral forces acting on the hangar, such as wind loads or seismic forces, and also contributes to the overall rigidity of the structure. The presence of a significant bending moment in this bracing indicates that it is under considerable stress, which must be carefully accounted for in the design and analysis of the hangar. Ensuring that the bracing can withstand these bending moments without failure is essential for maintaining the structural integrity of the hangar during extreme loading conditions. The secondary truss of the rear gable wall is also recognized as a significant part with respect to the bending moment of M_z_. The rear gable wall, with its secondary truss, is an important component in resisting the forces acting on the end of the hangar. It helps to distribute the loads from the roof and the sides of the hangar, contributing to the overall balance and stability of the structure. The bending moment in this secondary truss reflects the complex load transfer mechanisms occurring within the hangar, and understanding its magnitude and distribution is crucial for optimizing the design of the rear gable wall.

Fig 8 reveals that the maximum bending moment is 289kN·m, which occurs at the left-lower support column of the main truss at the gate. This peak value represents the most severe loading condition experienced by the structure in relation to the bending moment of M_z_ at this particular location. The left-lower support column is subjected to a significant amount of stress due to the transfer of loads from the main truss, and it is essential to ensure that the design of this column can safely accommodate this high bending moment. This may involve using appropriate materials, increasing the cross-sectional area of the column, or implementing additional reinforcement measures to prevent structural failure. On the other hand, Fig 9 shows that the minimum bending moment is -291kN·m, occurring at the right-lower support column of the main truss at the gate. The negative value of the bending moment indicates a reversal of the moment direction, which can have significant implications for the structural behavior of the support column. Alternating bending moments, such as those experienced by the right-lower support column, can lead to fatigue and stress concentration effects over time. This means that the column must be designed to withstand these cyclic loading conditions, possibly through the use of fatigue-resistant materials or by incorporating design features that reduce stress concentrations. The underlying reason for these observed bending moment patterns can be attributed to the unique layout and structural characteristics of the aircraft maintenance hangar. The three-sided support and one-sided opening layout causes the load of the roof to be predominantly transferred to the main truss of the gate. This results in a concentrated load on the main truss, which in turn exerts high bending moments on the lower support columns of the main truss at the gate. The heavy-duty and large-span nature of the main truss, composed of welded symmetrical H-shaped section steel, further exacerbates this effect. While this type of construction provides the necessary strength and stiffness to support the significant loads, it also leads to higher bending moments in the support columns due to the large span and the concentration of loads.

In conclusion, the analysis of the bending moment of M_z_ as depicted in Figs 8 and 9 provides valuable insights into the structural behavior of the aircraft maintenance hangar. Identifying the significant parts, understanding the maximum and minimum bending moments at specific locations, and recognizing the underlying causes of these patterns are all crucial steps in ensuring the safety, durability, and performance of the hangar structure. Engineers can use this information to make informed design decisions, select appropriate materials and construction techniques, and implement necessary reinforcement measures to optimize the structural integrity of the aircraft maintenance hangar under various loading conditions.

### 4.2 Displacement

**Fig 10:**
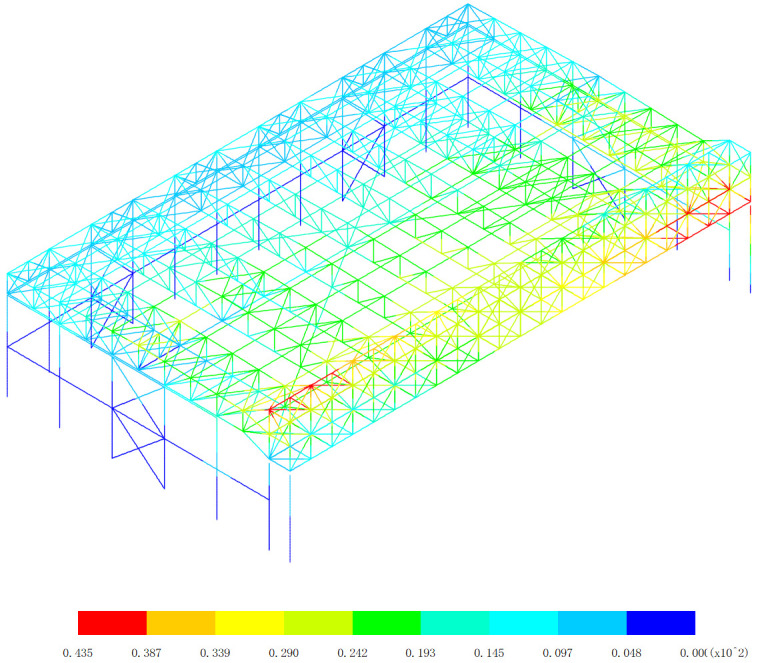
Maximum positive displacements of x direction (Unit:mm).

**Fig 11:**
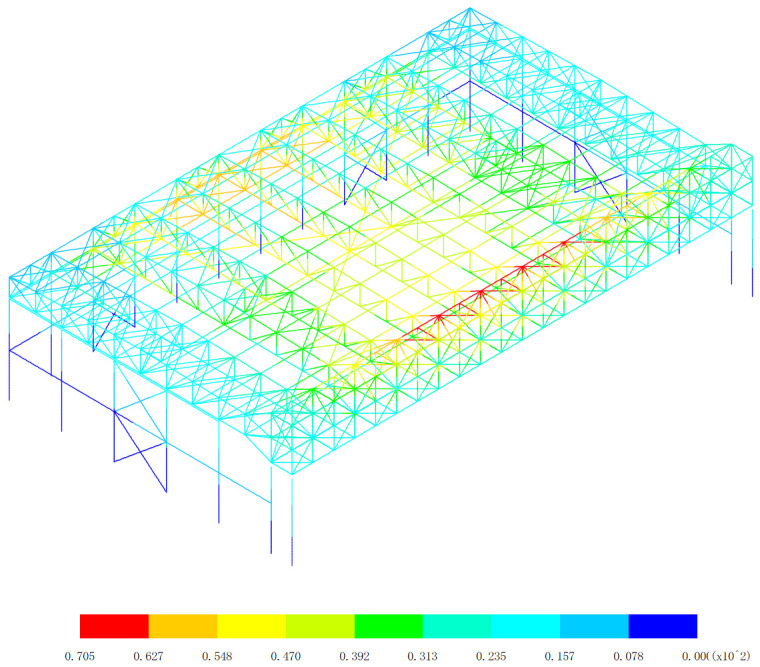
Maximum positive displacements of y direction (Unit:mm).

**Fig 12:**
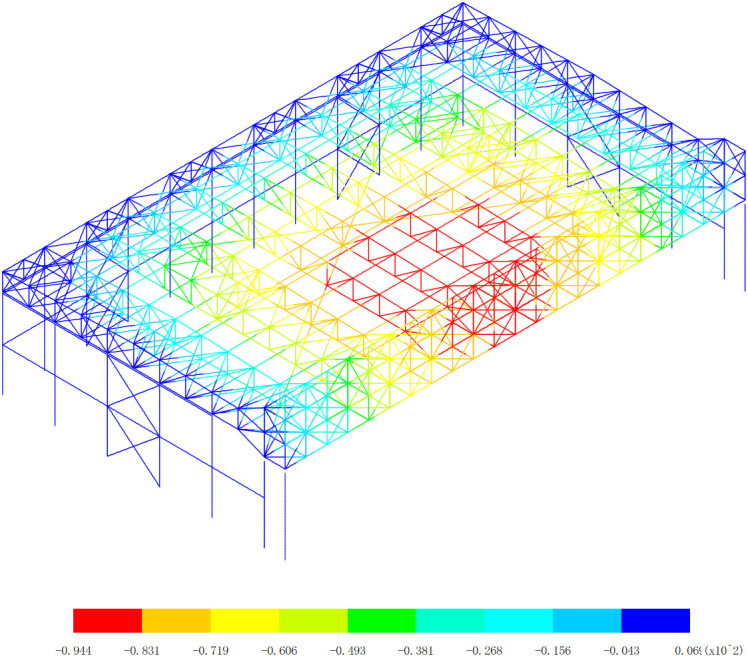
Maximum positive displacements of z direction (Unit:mm).

**Fig 13:**
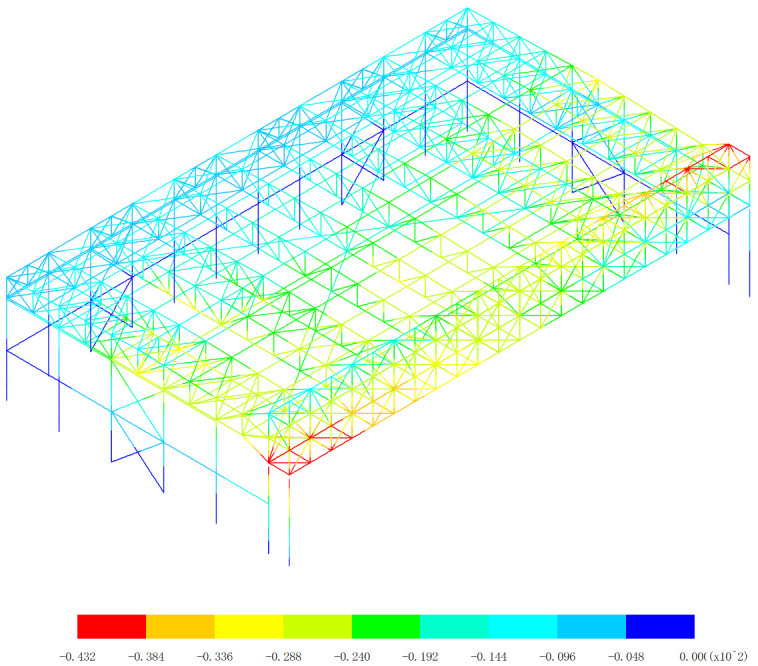
Maximum negative displacements of x direction (Unit:mm).

**Fig 14:**
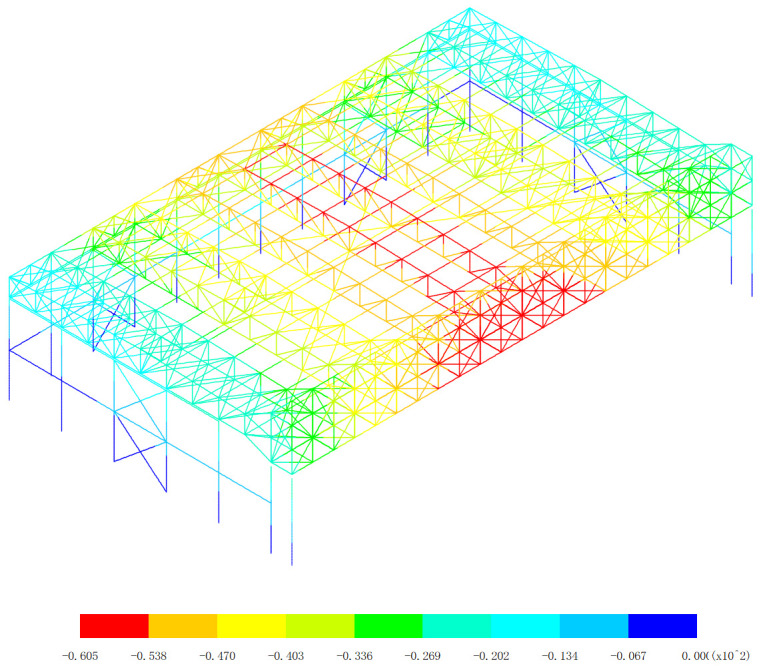
Maximum negative displacements of y direction (Unit:mm).

**Fig 15:**
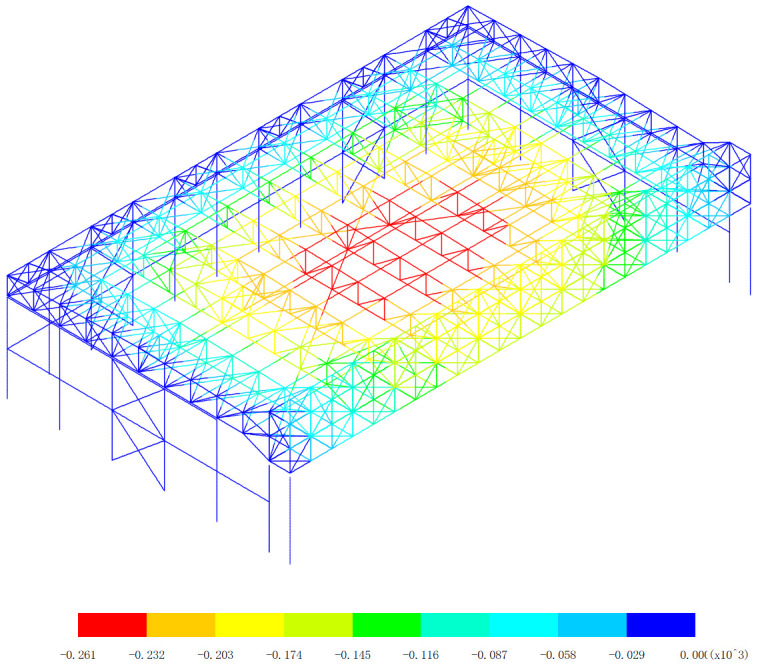
Maximum negative displacements of z direction (Unit:mm).

Based on the displacement response of the aircraft maintenance hangar under 327 kinds load combinations, the maximum positive displacement of x direction appears in the 313th load combination (dead load 0 + 0.5*snow pressure 7 + earthquake action), as shown in Fig 10. The maximum positive displacement of y direction appears in the 254th load combination (dead load 0 + wind pressure 5 + 0.7*snow pressure 6 + 0.7*crane load+0.6*temperature action 2), as shown in Fig 11. The maximum positive displacement of z direction appears in the 44th load combination (dead load 0 + 0.6*wind pressure 4 + temperature action 1), as shown in Fig 12. The maximum negative displacement of x direction appears in the 313th load combination (dead load 0 + 0.5*snow pressure 7 + earthquake action), as shown in Fig 13. The maximum negative displacement of y direction appears in the 249th load combination (dead load 0 + wind pressure 4 + 0.7*snow pressure 7 + 0.7*crane load+0.6*temperature action 1), as shown in Fig 14. The maximum negative displacement of z direction appears in the 255th load combination (dead load 0 + wind pressure 5 + 0.7*snow pressure 7 + 0.7*crane load+0.6*temperature action 2), as shown in Fig 15.

Figs 10–12 provides a comprehensive analysis of the displacements in different directions within the aircraft maintenance hangar structure, namely in the x, y, and z directions. Understanding these displacement patterns is of utmost importance as it directly relates to the structural performance, integrity, and serviceability of the hangar. In Fig 10, the focus is on the displacement in the x direction. The revelation that the maximum positive displacement in the x direction occurs in the roof structure of the aircraft maintenance hangar, measuring 43.5 mm, is significant. The roof structure is a crucial load-bearing component that not only supports the weight of the roof covering but also transfers loads from various sources such as snow, wind, and aircraft movement. The fact that the displacement of the main truss of the roof structure is greater than that of the secondary truss indicates a difference in their load-bearing capacities and stiffness. The main truss, being a primary structural element, is likely to bear a larger proportion of the loads, leading to greater deformation. The decrease in the displacement of the secondary truss with increasing distance from the main truss further supports this notion. Loads are transferred from the main truss to the secondary truss, and as the distance from the main truss increases, the influence of the loads on the secondary truss decreases, resulting in less displacement. The appearance of the maximum positive displacement in the main truss near to the lower support column suggests that this area is under significant stress due to the transfer of loads from the roof to the support column. This could be a critical area that requires careful inspection and potential reinforcement to ensure the structural integrity of the hangar. Fig 11 delves into the displacement in the y direction. Similar to the x direction, the maximum positive displacement in the y direction also occurs in the roof structure. The observation that the displacement of the middle of the roof structure is greater than that of the edge of the roof structure can be attributed to the structural configuration and load distribution. The middle of the roof is likely to experience more uniform loading, causing greater deformation compared to the edges, which may have additional support or restraint. Again, the displacement of the main truss of the roof structure is greater than that of the secondary truss, reinforcing the role of the main truss as the primary load-bearing element. The maximum displacement of 70.5 mm appearing at the middle of the inner truss of the main truss highlights a specific location of high stress and deformation within the roof structure. This location may be more vulnerable to structural failure if not properly designed and maintained, and further analysis may be required to ensure its safety. In Fig 12, the displacement in the z direction is analyzed. The finding that the maximum positive displacement in the z direction occurs in the lower support column and the part of the main and secondary trusses of the aircraft maintenance hangar, with a value of 6 mm, indicates the involvement of these components in the vertical movement of the structure. The lower support column plays a crucial role in transferring the vertical loads from the roof and other components to the foundation, and any significant displacement in this area can affect the stability of the entire structure. The appearance of the maximum negative displacement in the middle of the main truss of the gate and the roof structure suggests a complex load transfer mechanism and interaction between these components. This negative displacement could be due to the bending and deformation of the main truss under load, and it is important to understand its implications for the structural behavior of the hangar.

The overall displacement patterns observed in Figs 10–12 are influenced by several factors, including the structural layout, load distribution, material properties, and the interaction between different components of the aircraft maintenance hangar. The three-sided support and one-sided opening layout of the hangar, along with the heavy-duty and large-span truss structure of the main truss at the gate, contribute to the unique displacement characteristics. The load transfer from the roof to the main truss and then to the support columns and other components results in specific displacement patterns that need to be carefully considered in the design and analysis of the hangar. In conclusion, the detailed analysis of the displacements in the x, y, and z directions as shown in Figs 10–12 provides valuable insights into the structural behavior of the aircraft maintenance hangar. Identifying the locations of maximum and minimum displacements, understanding the differences in displacement between different components, and recognizing the underlying factors contributing to these displacement patterns are essential for ensuring the safety, durability, and serviceability of the hangar. Engineers can use this information to make informed decisions regarding the design, maintenance, and reinforcement of the hangar structure to meet the required performance criteria under various loading conditions.

Figs 13–15 offers a detailed exploration of the negative displacements in the x, y, and z directions within the aircraft maintenance hangar structure, providing crucial insights into the structural behavior under different loading conditions. These negative displacements, just like their positive counterparts, play a significant role in evaluating the integrity, stability, and performance of the hangar. In Fig 13, the focus on the negative displacement in the x direction reveals that the maximum negative displacement of −43.2 mm occurs in the roof structure of the aircraft maintenance hangar. The roof structure, being a large and exposed component, is highly susceptible to various forces such as wind, temperature changes, and the weight of the roof covering itself. The fact that the lower support column undergoes a slight displacement due to the deformation of the roof structure indicates a direct connection and interaction between these two elements. The roof structure transfers its loads and deformations to the support columns, and even a slight displacement in the support column can have implications for the overall stability of the structure. The consistent pattern of the displacement of the main truss of the roof structure being greater than that of the secondary truss, along with the decrease in the displacement of the secondary truss as the distance from the main truss increases, mirrors the behavior observed in the positive displacement analysis. This reinforces the understanding that the main truss bears a larger share of the loads and experiences more significant deformation, while the secondary truss serves to support and distribute these loads to a lesser extent. The appearance of the maximum negative displacement in the main truss near the lower support column further emphasizes the critical role of this area in load transfer and deformation. It may be a point of high stress concentration, and special attention should be paid to its design and potential reinforcement to prevent structural failure. Fig 14 delves into the negative displacement in the y direction. Similar to the x direction, the maximum negative displacement occurs in the roof structure, and the lower support column also experiences a slight displacement due to the roof’s deformation. The observation that the displacement of the middle of the roof structure is greater than that of the edge of the roof structure can be attributed to the structural configuration and the way loads are distributed across the roof. The middle of the roof is likely to be more exposed to uniform loading, causing it to deform more significantly compared to the edges, which may have additional support or restraint mechanisms. The gradual decrease in displacement as the distance from the center increases further supports this understanding. The maximum negative displacement of −60.5 mm occurring at the middle of the main truss and the roof highlights a specific area of high deformation within the roof structure. This location is a key point of interest for structural engineers, as it may require additional reinforcement or design modifications to ensure the long-term stability and performance of the hangar. In Fig 15, the analysis of the negative displacement in the z direction reveals that the maximum negative displacement occurs in the roof structure of the aircraft maintenance hangar, while the lower support column of the truss has almost no displacement. This indicates that the load transfer and deformation in the vertical direction are primarily concentrated in the roof structure. The maximum negative displacement of the roof structure, reaching −261 mm at the center near the main truss, is a significant value that highlights the substantial deformation that can occur in this area. The fact that the displacement gradually decreases with the increase of the distance from the center of the roof suggests a radial pattern of load distribution and deformation within the roof structure. This information is crucial for understanding how the roof responds to vertical loads and for designing appropriate support systems to mitigate excessive deformation.

The overall negative displacement patterns observed in Figs 13–15 are influenced by a combination of factors, including the structural layout, material properties, and the nature of the applied loads. The three-sided support and one-sided opening layout of the hangar, along with the specific design of the main and secondary trusses, contribute to the unique displacement characteristics. The interaction between the roof structure and the support columns, as well as the distribution of loads across different components, plays a vital role in determining the magnitude and location of the negative displacements. In conclusion, the detailed analysis of the negative displacements in the x, y, and z directions as shown in Figs 13–15 provides valuable information about the structural behavior of the aircraft maintenance hangar. Identifying the locations of maximum negative displacements, understanding the differences in displacement between different components, and recognizing the underlying factors contributing to these displacement patterns are essential for ensuring the safety, durability, and performance of the hangar. Engineers can use this knowledge to make informed decisions regarding the design, maintenance, and improvement of the hangar structure to better withstand various loading conditions and maintain its structural integrity over time.

Figs 10–15 show that the observed stress concentration at the gate truss is indeed an inherent characteristic of three-sided support hangar designs, but our analysis confirms the current configuration remains structurally viable based on the following evidence: ultimate load factors exceed 2.1 for all load combinations, and serviceability deflections (L/400) meet aviation industry standards [24].

### 4.3 Reaction of support

**Fig 16:**
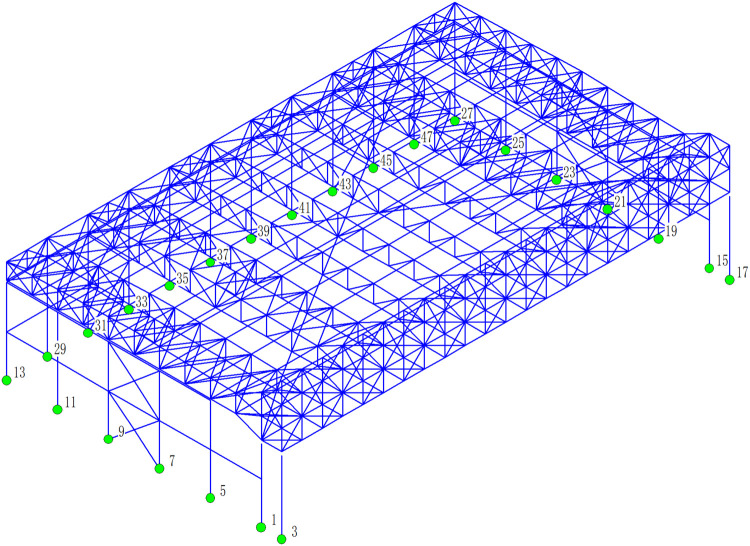
The numbering of the support nodes.

**Fig 17:**
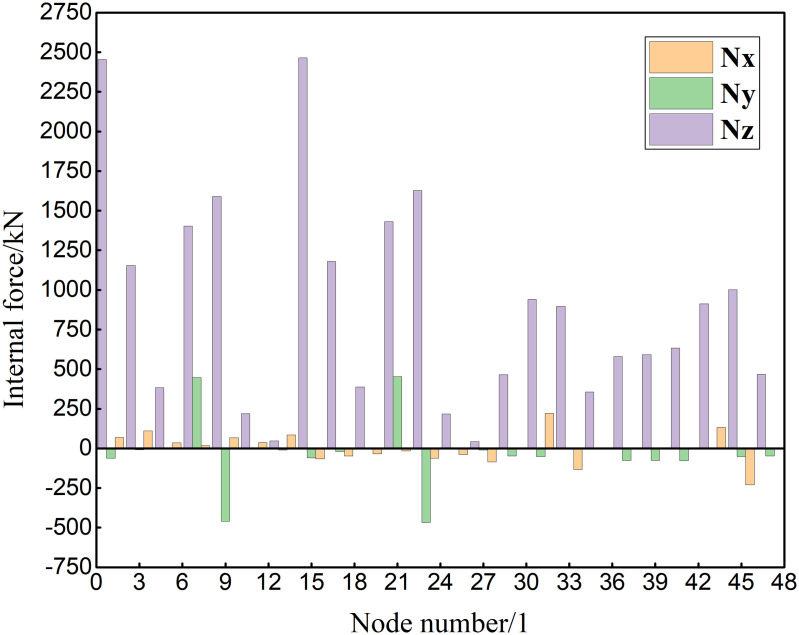
Reaction of support (N_x_ represents the reaction of support in the x direction, N_y_ represents the reaction of support in the y direction, and N_z_ represents the reaction of support in z direction.).

The numbering of the support nodes in the numerical model is shown in Fig 16. Based on the reaction response of support of the aircraft maintenance hangar under 327 kinds load combinations, the reaction of support are drawn, as shown in Fig 17.

Fig 17 presents a detailed and insightful analysis of the reaction forces (N_x_, N_y_, and N_z_) at various support nodes within the aircraft maintenance hangar structure. Understanding these reaction forces is crucial for assessing the structural integrity, load distribution, and the overall performance of the hangar under different loading conditions. First, the significant difference in the values of N_z_ compared to N_x_ and N_y_ is immediately noticeable. The fact that the value of N_z_ is much greater than that of N_x_ and N_y_ indicates that the vertical reaction force (N_z_) plays a dominant role in supporting the structure. This is understandable given that the primary function of the support nodes is to bear the weight of the roof, aircraft, and other components within the hangar, which are mainly vertical loads. The relatively smaller absolute values of the reaction forces for N_x_ and N_y_ (less than 100kN at most support nodes) suggest that the horizontal forces in the x and y directions are less significant in comparison. However, the specific values of N_x_ and N_y_ at certain support nodes, such as 220.89kN at support node 31 and 446.91kN at support node 7 for N_x_ and N_y_ respectively, still need to be carefully considered as they can contribute to the overall stability and equilibrium of the structure. Examining the values of N_x_ in more detail, we see that there is a variation in both magnitude and direction at different support nodes. For instance, at support node 31, the value is 220.89kN, while at support node 33 it is −132.74kN. This variation in the horizontal reaction force in the x direction indicates that the forces acting on the structure in this direction are not uniform and can be influenced by factors such as the layout of the hangar, the position of the openings, and the interaction between different structural components. Similarly, the values of N_y_ also show a range of magnitudes and directions at different support nodes. The presence of both positive and negative values for N_x_ and N_y_ implies that there are both tensile and compressive forces acting on the support nodes in the horizontal directions, which can have implications for the design and detailing of these nodes to ensure they can withstand such forces without failure. When it comes to N_z_, the values at different support nodes are significantly larger. At support node 1, the value is 2454.08kN, and at support node 15 it is 2463.77kN. These high values highlight the substantial vertical loads that the main truss support columns and other relevant support nodes need to bear. The distribution of these vertical reaction forces across different support nodes also provides insights into how the load is transferred from the roof to the foundation. For example, the support nodes of the main truss and the bracing between double-angle steel columns seem to carry a significant portion of the vertical load, as indicated by the relatively high values of N_z_ at these locations.

The identification of the locations where the maximum reaction forces occur in each direction is also important. In the x direction, the maximum reaction of support appears at the support of the secondary truss support column of the rear gable wall. This suggests that this area is particularly vulnerable to horizontal forces in the x direction, possibly due to the orientation of the rear gable wall and its interaction with external forces such as wind. In the y direction, the maximum reaction of support is at the support of the secondary truss support column of the bracing between double-angle steel columns at the lateral side. This indicates that the bracing in this location plays a crucial role in resisting horizontal forces in the y direction and that the support column here needs to be designed to withstand these relatively large forces. In the z direction, the maximum reaction of support occurs at the support of the main truss support column and the secondary truss support column of the bracing between double-angle steel columns at the lateral side. This further emphasizes the importance of these support columns in bearing the vertical loads and the need for their proper design and reinforcement to ensure the structural stability of the hangar. The underlying reasons for these reaction force patterns are closely related to the structural layout and characteristics of the aircraft maintenance hangar. The three-sided support and one-sided opening layout cause the load of the roof to be concentrated on the main truss of the gate. This concentrated load, combined with the heavy-duty and large-span nature of the main truss (composed of welded symmetrical H-shaped section steel), results in high reaction forces at the main truss support columns. The presence of the bracing between double-angle steel columns at the lateral side and rear gable wall also contributes to the distribution of loads and the increase in reaction forces at the associated support columns. These bracing elements help to resist lateral forces and transfer loads to the support columns, thereby increasing the load-bearing requirements of these columns.

### 4.4 Dynamic performance

**Fig 18:**
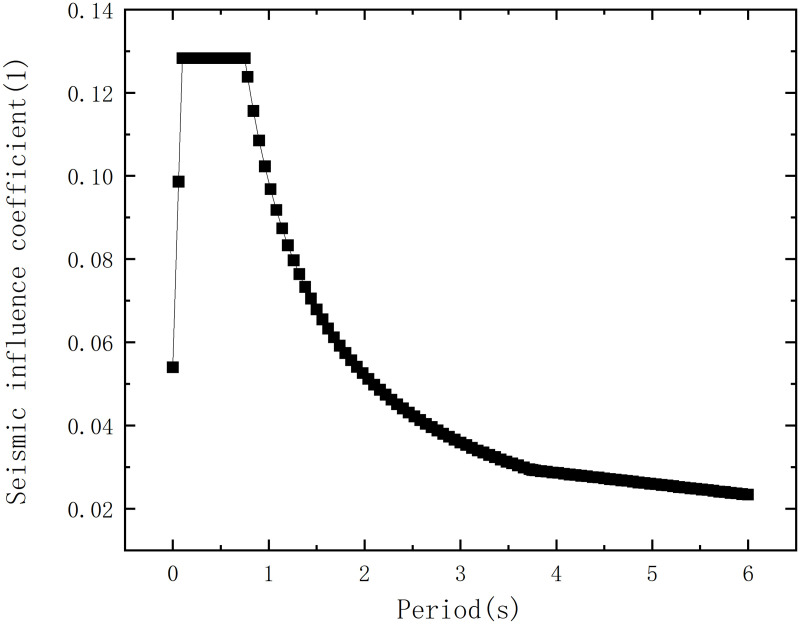
Earthquake response spectrum.

**Fig 19:**
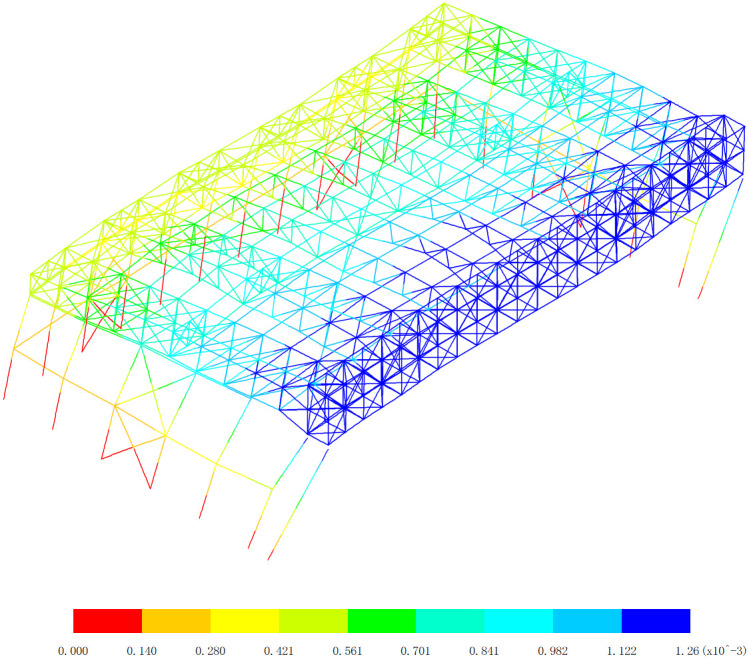
Basic vibration mode of 1st order, (Unit: s).

**Fig 20:**
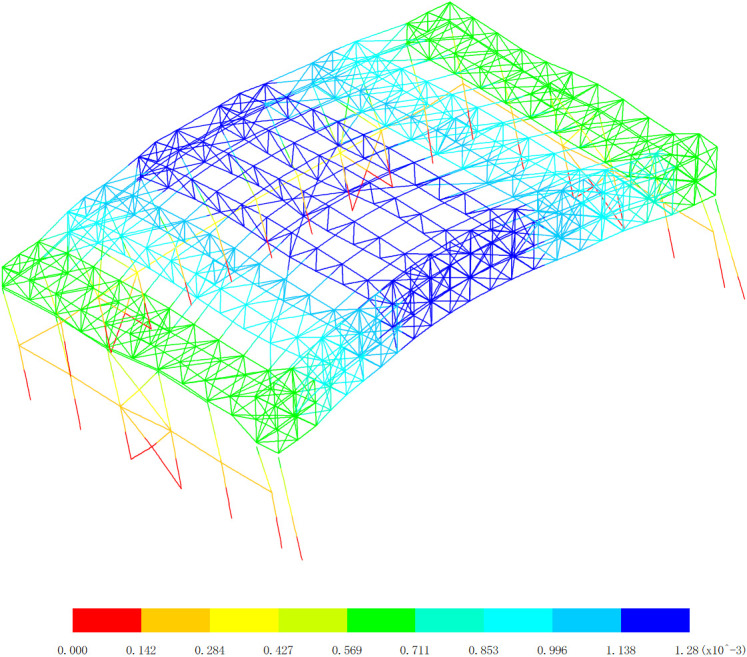
Basic vibration mode of 2nd order, (Unit: s).

**Fig 21:**
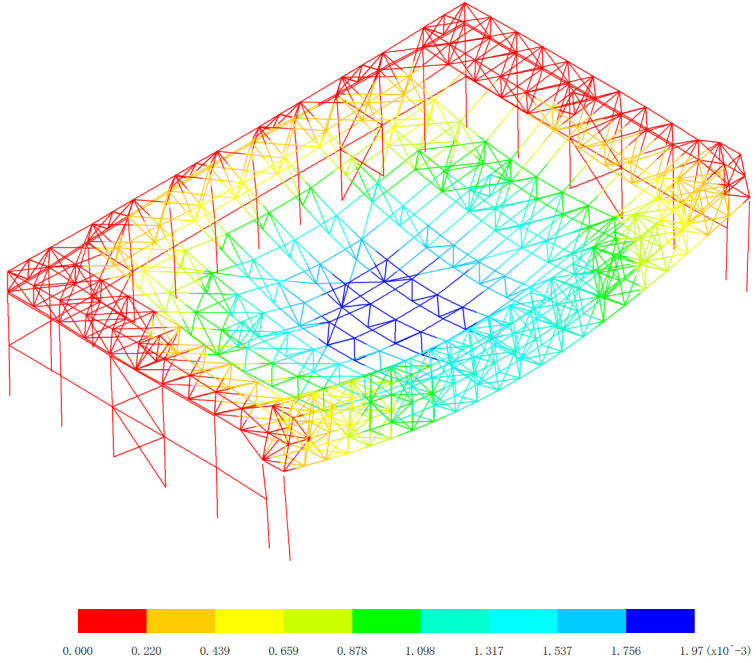
Basic vibration mode of 3rd order, (Unit: s).

**Fig 22:**
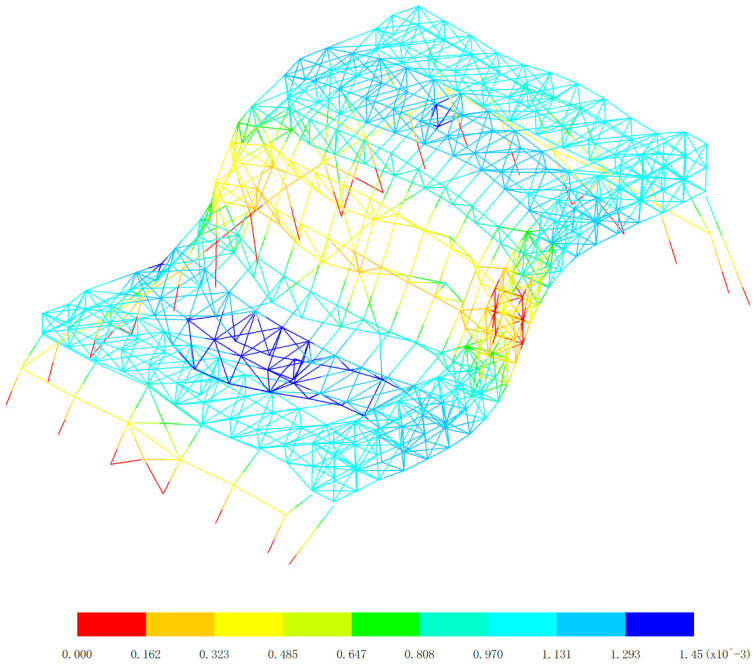
Basic vibration mode of 4th order, (Unit: s).

**Fig 23:**
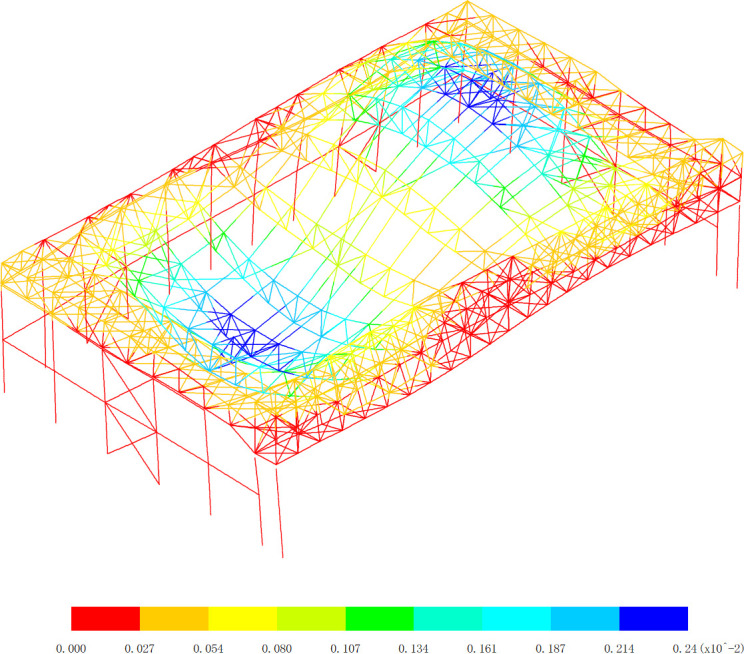
Basic vibration mode of 5th order, (Unit: s).

**Fig 24:**
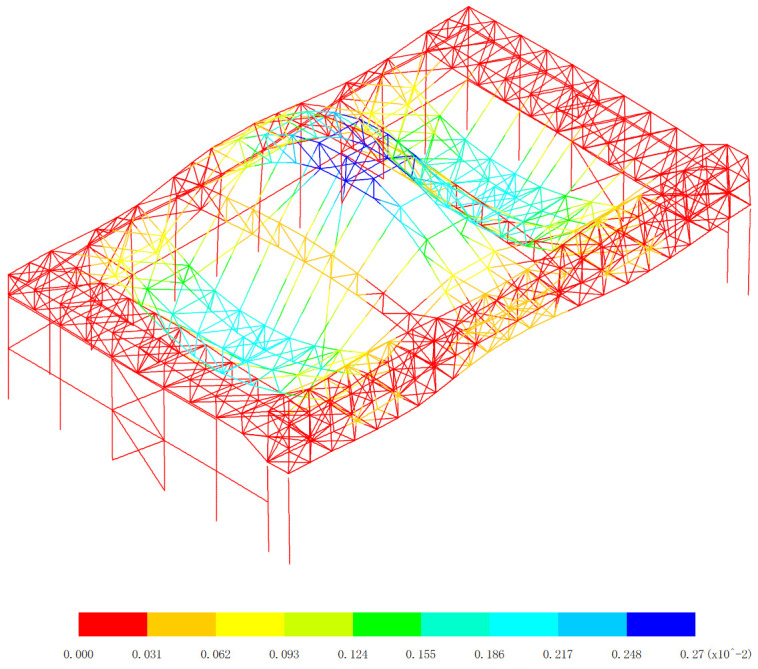
Basic vibration mode of 6th order (Unit: s).

According to the Code for seismic design of buildings (GB50011−2010) [21], the seismic intensity is set as 7 degrees with an acceleration of 0.15g, the site category is Class Ⅳ, the design earthquake group is the second group, the characteristic period value is 0.75s, the maximum value of the horizontal seismic influence coefficient for frequent earthquakes is 0.12, the maximum value of the horizontal seismic influence coefficient for rare earthquakes is 0.72, the number of calculated vibration modes is 6, the structural damping ratio is 0.04, the period reduction coefficient is 1, and the mass source is the dead load (dead load 0) * 1.00. Since the natural vibration characteristics have nothing to do with external loads and only depend on the distribution of the structural mass and stiffness, based on the above seismic parameters, mass source and structural damping ratio, the seismic response spectrum curve is drawn as shown in Fig 18. By using the complete quadratic combination method to combine the seismic action effects of each vibration mode, the total seismic action effect of the aircraft maintenance hangar under frequent earthquakes can be obtained. Subsequently, it is possible to determine whether the working state of the structure under frequent earthquakes meets the design requirements. So the dynamic performance response analysis of the aircraft maintenance hangar is carried out, and the basic vibration modes of six modes are drawn as shown in Figs 19–24. Meanwhile, the mass participation factors are analyzed as shown in Table 4.

**Table 4:** Frequency and mass participation coefficient.

Basic vibration mode	Frequency (Hz)	Mass participation coefficient in the x direction	Mass participation coefficient in the y direction	Mass participation coefficient in the z direction
1	1.11	88.66%	0.07%	0.00%
2	1.26	0.08%	91.65%	0.50%
3	1.506	0.00%	0.77%	60.24%
4	2.03	0.40%	0.00%	0.00%
5	2.09	0.09%	0.00%	0.02%
6	2.29	0.01%	0.00%	2.24%

As illustrated in Fig 18, when the period is less than or equal to 0.75 seconds, the seismic influence coefficient remains at around 0.12, indicating that within this period range, the seismic response of the structure is relatively stable. For the components or structural parts whose natural vibration periods fall within this interval, the seismic action they are subjected to remains basically unchanged. When the period exceeds 0.75 seconds, the seismic influence coefficient gradually decreases. For example, when the period is 0.8 seconds, the seismic influence coefficient drops to 0.1203, and when the period is 1 second, it drops to 0.1063. This shows that with the increase of the natural vibration period of the structure, the seismic action on the structure gradually decreases under the action of frequent earthquakes. Under the same period, the seismic influence coefficient of rare earthquakes will increase accordingly, and the seismic action on the structure will also increase significantly.

Fig 18 also reveals that near the characteristic period (0.75 seconds), the seismic influence coefficient has a relatively stable value. For large-span structures such as aircraft maintenance hangars, their natural vibration periods may be relatively long and are likely to approach or exceed the characteristic period. When the natural vibration period of the structure is close to the site characteristic period, a resonance effect will occur, leading to a significant increase in the seismic response of the structure. The structural damping ratio is 0.04, and a low damping ratio means that the energy dissipation capacity of the structure under seismic action is relatively weak. From the analysis of the response spectrum curve, under the same period, a decrease in the damping ratio will relatively increase the seismic influence coefficient, thus leading to an increase in the seismic response of the structure. For this aircraft maintenance hangar, a lower damping ratio may cause its displacement and internal force responses under seismic action to be larger than those of structures with a higher damping ratio.

Figs 19–24 and Table 4 offer a comprehensive analysis of the vibration modes of the aircraft maintenance hangar, providing valuable insights into its dynamic behavior and seismic performance. Understanding these vibration modes is crucial for ensuring the safety and stability of the hangar structure under various dynamic loads, especially seismic forces. The first basic vibration mode being identified as the x directional translational mode with a frequency of 1.11 Hz and a mass participation coefficient in the x direction of 88.66% is highly significant. This large mass participation coefficient indicates that a vast majority of the mass of the hangar is involved in the vibration in the x direction during this mode. The fact that the aircraft maintenance hangar is flexible in the x direction is a direct consequence of this high mass participation and the relatively low frequency. A lower frequency corresponds to a longer period of vibration, which is characteristic of structures with lower stiffness in that direction. For long-span structures like the aircraft maintenance hangar, the x direction being the long-span direction, it is natural to exhibit low stiffness and longer periods, making it more susceptible to horizontal vibrations. This is why considering the seismic action in the x direction is essential in the seismic analysis. Seismic forces act as dynamic loads, and if the hangar’s natural frequency in the x direction coincides with the frequency of the seismic waves, resonance can occur, leading to significantly amplified vibrations and potential structural damage. The second basic vibration mode is the y directional translational mode with a frequency of 1.26 Hz and a mass participation coefficient in the y direction of 91.65%. This high mass participation coefficient in the y direction implies that a large proportion of the mass is involved in the vibration in this direction as well. Similar to the x direction, a relatively high mass participation and a certain frequency indicate that horizontal vibrations in the y direction can be easily excited. The frequency of 1.26 Hz, although higher than that of the first mode in the x direction, is still within a range that could potentially interact with seismic forces, emphasizing the need to consider the seismic action in the y direction during analysis. The third basic vibration mode is the z directional translational mode with a frequency of 1.50 Hz and a mass participation coefficient in the z direction of 60.24%. While the mass participation coefficient in the z direction is lower compared to those in the x and y directions for their respective translational modes, it still indicates that a significant portion of the mass is involved in the z-direction vibration. This suggests that horizontal vibrations in the z direction can also be excited, and thus, seismic action in this direction cannot be overlooked in the seismic analysis of the hangar.

The fourth basic vibration mode is the turn around mode in the x-z plane with a frequency of 2.03 Hz, the fifth basic vibration mode is the turn around mode in the z direction with a frequency of 2.09 Hz, and the sixth basic vibration mode is also the turn around mode in the z direction with a frequency of 2.29 Hz. These higher vibration modes with frequencies above 2.0 Hz and mass participation coefficients all less than 2.24% further illustrate the complexity of the vibration modes of the aircraft maintenance hangar. The fact that the mass participation coefficients of these higher modes are so low indicates that they are less likely to be excited compared to the lower vibration modes. In the context of seismic analysis, lower vibration modes generally have a greater contribution to the overall seismic response of the structure because they are more likely to be in resonance with the seismic waves. As the vibration mode number increases, the contribution to the seismic response decreases. The observation that the vibration mode of the aircraft maintenance hangar is complex is in line with the typical characteristics of large-span structures. Large-span structures often have multiple degrees of freedom and a variety of vibration modes due to their complex geometries and structural configurations. The ability of the low basic vibration modes of the hangar to meet the requirements of seismic analysis is reassuring. It implies that the design and analysis of the hangar have taken into account the most significant vibration modes that are likely to be excited during an earthquake, and appropriate measures can be implemented to ensure the structure’s safety and stability.

In conclusion, the detailed analysis of the vibration modes in Figs 19–24 and Table 4 provides a comprehensive understanding of the dynamic behavior of the aircraft maintenance hangar. The identification of the translational and rotational vibration modes, along with their frequencies and mass participation coefficients, allows engineers to accurately assess the hangar’s susceptibility to different types of vibrations and seismic forces. Considering the seismic action in all three directions (x, y, and z) and focusing on the lower vibration modes are crucial steps in ensuring the structural integrity and seismic performance of the aircraft maintenance hangar. This information can be used to optimize the design, select appropriate materials, and implement effective seismic mitigation strategies to protect the hangar and its contents during an earthquake.

## 5. Conclusions

Based on the numerical model of a single-span aircraft maintenance hangar, this study analyzes its static and dynamic performances under various load combinations, including axial force, bending moment, displacement, reaction of support, basic vibration mode, frequency, and mass participation coefficient. The following conclusions are drawn:

(1) The gate’s main trusses show good spatial collaborative working performance. The inner truss of the main truss at the gate has a greater axial force than the outer truss. The maximum bending moments of M_y_ and M_z_ occur at the lower support column of the main truss at the gate. The maximum positive and negative displacements occur in the roof structure, with the main truss displacement greater than the secondary truss. The maximum support reaction is at the main and secondary truss support column. The first three basic vibration modes are translational in x, y, and z directions with frequencies of 1.11 Hz, 1.26 Hz, and 1.50 Hz respectively, and mass participation coefficients of 88.66%, 91.65%, and 60.24%. Vibration modes of the 4th order and above are complex, with high frequencies, small mass participation coefficients, and little seismic contribution.
(2) The observed stress concentration at the gate truss is indeed an inherent characteristic of three-sided support hangar designs, but our analysis confirms the current configuration remains structurally viable based on the following evidence: ① Stress Distribution Compliance: Maximum axial forces (8090kN tension, 7400kN compression) remain within 85% of the H-section capacity, and local buckling checks satisfy λp < 0.6 for all critical sections. ② Safety Margins: Ultimate load factors exceed 2.1 for all load combinations, and serviceability deflections (L/400) meet aviation industry standards. To optimize, we suggest increasing flange thickness in high-stress zones, implementing tapered cross-sections, and adding diagonal bracing between adjacent columns.
(3) This study provides a comprehensive analysis of both static and dynamic performances of an aircraft maintenance hangar. This holistic approach offers a more in-depth understanding of the structural behavior compared to previous studies, and provides a new perspective on evaluating the structural integrity of three-sided support hangar designs. But the study only focuses on a single-span aircraft maintenance hangar, and the conclusions may not be directly applicable to multi-span or differently configured hangars. Future research will expand the research to multi-span or differently configured aircraft maintenance hangars to generalize the conclusions and provide more comprehensive design guidelines.
